# Analysis of CDR3 region diversity with different lengths in bovine immunoglobulin heavy chain genes

**DOI:** 10.3389/fimmu.2025.1722984

**Published:** 2025-12-29

**Authors:** Haidong Zhao, Xiaoqin Tang, Yuelang Zhang, Mingli Wu

**Affiliations:** 1 Guangxi Key Laboratory of Brain and Cognitive Neuroscience, Guilin Medical University, Guilin, China; 2 College of Medical Artificial Intelligence, Guilin Medical University, Guilin, China; 3 Key Laboratory of Molecular Medical Engineering, Guilin Medical University, Guilin, China; 4 College of Agriculture and Animal Husbandry, Qinghai University, Xining, Qinghai, China; 5 Hainan Institute of Zhejiang University, Sanya, Hainan, China

**Keywords:** bovine, immunoglobulin heavy chain, expression diversity, complementarity determining region 3 length, race

## Abstract

**Introduction:**

Cattle produce a unique antibody repertoire characterized by an exceptionally wide range of complementarity determining region 3 heavy chain (CDR3H) lengths, spanning from 1 bp to 204 bp—a feature that is extremely rare among mammals. The diversity characteristics of CDR3 segments of varying lengths and the underlying genetic and structural mechanisms remain an active area of research.

**Methods:**

We constructed CDR3 expression libraries from splenic tissues of eight adult cattle using RACE-PCR, followed by high-throughput sequencing. CDR3 regions were analyzed statistically in accordance with IMGT standards and structural characteristics of immunoglobulins.

**Results:**

High-throughput sequencing yielded a total of 473,067 high-quality reads. The CDR3 regions exhibited a broad length distribution, with the maximum reaching 234 bp. Based on density stacking analysis, CDR3 lengths were classified into four distinct groups: short (1~30 bp; 11.91%~16.93%), normal (31~100 bp; 81.51%~85.02%), long (101~150 bp; 0.12%~0.30%), and ultra-long (>150 bp; 0.16%~2.76%). Based on IMGT standards analysis, non-templated (N) nucleotides is the primary factor influencing CDR3 length. Each group displayed distinct preferences in V and D gene segment usage. The short CDR3 group was dominated by V1–14 and V1-39, the normal group was characterized predominantly by V1-39, while the long and ultra-long groups showed a strong preference for V1-7. Cattle possess two IgM subtypes, IgM1 and IgM2. The CDR3 length of the IgM1 subtype is primarily distributed in the short and normal groups. Within the normal CDR3 group, IgM1 exhibits a strong preference and exceptionally high utilization (70~80%) for the D4–1 gene segment. This stands in stark contrast to the usage of other D gene segments, particularly D8-2, which participates in CDR3 formation at a much lower frequency of merely 5~20%.

**Discussion:**

In summary, our findings suggest that the diversification of bovine CDR3H length is a multifactorial process. It results not only from biased V(D)J recombination but is also influenced by the balance between exonuclease and TdT activities, specialized subregions within germline gene segments, N/P nucleotide insertions, stochastic trimming of gene ends, and the formation of unique structural motifs such as disulfide bonds. These findings provide a foundational model for understanding the architecture and generation of complex antibody repertoires.

## 1 Introduction

Antibodies are key molecules of the adaptive immune system, capable of specifically recognizing and neutralizing an almost limitless variety of pathogens. This remarkable recognition ability stems from the immense diversity of the antibody variable region, particularly the complementarity determining regions (CDRs) (1). Among these, the CDR3 region, serving as the core of the antibody binding site, plays an irreplaceable role in diversity generation, antigen recognition, and clinical applications (2). The generation of antibody diversity is a complex, multi-stage process involving multiple mechanisms, occurring primarily during B cell development and activation, including combinatorial diversity, junctional diversity, somatic hypermutation, and class switch recombination (3–7).

Structurally, the heavy and light chains of the antibody variable region each possess three hypervariable loops, known as CDRs (CDR1, CDR2, CDR3), which collectively form the antigen-binding site. The CDR3H is the most diverse and structurally critical segment among the three CDRs (8). CDR3H is encoded by the junction of the V-(D)-J gene segments, spanning the V-D and D-J regions that are rich in junctional diversity, accounting for its status as the loop with the highest sequence variability and paramount spatial structural importance. In the three-dimensional structure, CDR3H is typically located at the center of the antigen-binding pocket, directly engaging in the closest contact with the antigenic epitope (9, 10). Therefore, it is often regarded as the “structural fingerprint” determining antibody binding specificity. Although the general mechanisms of antibody diversity formation-such as V(D)J recombination, junctional diversity, and somatic hypermutation (SHM)-are relatively conserved across species, the immune system of cattle (Bos taurus) has evolved a set of unique and efficient strategies. The bovine immunoglobulin gene repertoire exhibits a phenomenon of “simplification” (limited number of V/D/J genes) coexisting with “specialization” (the presence of ultralong DH genes) (11, 12). By shifting the focus of diversity generation from germline complexity to the extreme diversification of somatic mutations, especially within the CDR3H region, cattle construct their vast antibody repertoire in a highly efficient manner (13, 14). The most prominent feature is that a subset of antibodies possesses ultralong CDR3Hs, which can reach lengths of up to 70 amino acids. In contrast to the restricted length distribution of human and mouse CDR3H (typically 8~16 amino acids), the bovine CDR3H repertoire displays a vast length span, encompassing a hallmark “ultra long” subgroup that is extremely rare in other known species (15, 16).

These unique structural characteristics are well-documented; however, the driving factors behind the “structural explosion from limited combinatorial building blocks” model remain contentious. During early B cell development, activation-induced deaminase (AID)-driven somatic mutation operates in a manner akin to “directed evolution” generating or removing cysteines en masse within the “knob” domain, thereby dynamically creating, breaking, or remolding different disulfide bond pairings (17, 18). Some studies suggest that unique gene sequences and rearrangement mechanisms are important reasons for the large span of CDR3 length in bovine. (18–20). All ultralong CDR3Hs originate from a specific germline VH gene (VH1-7) and an ultralong DH gene (DH2) (18, 21). VH1–7 encodes the base of the “stalk” structure and its critical interface for pairing with a constant light chain, while DH2 provides the genetic template encoding the descending strand of the “stalk” and the core of the “knob” domain (18, 21, 22). Atypical, extensive nucleotide insertions during the V-D-J rearrangement process are also considered a major driver of bovine immunoglobulin diversity. However, the core diversity engine is believed to be somatic hypermutation centered on cysteine. Although deep sequencing of the bovine antibody repertoire has revealed significant diversity of cysteine residues within ultralong CDR3 regions, the analysis of unconventional CDR3 lengths in cattle remains incomplete due to the dominance of conventional-length CDR3s in abundance, which overshadows other CDR3 segment lengths. (17, 23).

Therefore, while the bovine antibody repertoire has been noted for its extraordinary CDR3 length diversity, a comprehensive and systematic understanding of its full architectural spectrum has been lacking. Previous investigations have often centered on the singular phenomenon of ultra-long CDR3s, leaving the fundamental organizational principles that govern the entire repertoire poorly defined. To address this gap, the primary objective of this study was to establish a holistic architectural model of the naïve bovine IgM repertoire by systematically defining its complete CDR3 length spectrum. We specifically aimed to decipher the distinct molecular signatures—including VDJ recombination preferences and the contribution of junctional diversification mechanisms—that differentiate CDR3 regions of different lengths. A critical and novel aspect of our investigation was to evaluate the potential functional specialization between the IgM1 and IgM2 isotypes in generating this repertoire, a question that has remained largely unexplored. By resolving these questions, this work provides a foundational framework that shifts the paradigm from studying ultra-long CDR3s in isolation to understanding the bovine antibody repertoire as a strategically organized and highly compartmentalized system.

## 2 Materials and methods

### 2.1 Spleen collection and RNA extraction

Spleen tissues were collected from eight healthy domestic cattle at a slaughterhouse in Yangling, Shaanxi Province. Total RNA was extracted using the TRIzol methodv from 200 mg spleen tissue, quantified on a NanoDrop 1000 spectrophotometer, and assessed for integrity by agarose gel electrophoresis. Qualified RNA samples were stored at -80°C until further use. All experimental procedures were approved by the Institutional Animal Care and Use Committee of Guilin Medical University (Approval No. GLMC202503345) and conducted in accordance with the Regulation on the Administration of Laboratory Animals (2017 Revision) issued by the State Council of China.

### 2.2 5’-RACE-Ready cDNA synthesis and CDR3H repertoire construction

5’-RACE-Ready cDNA was synthesized from total RNA using the SMARTer^®^ RACE 5’/3’ Kit (Takara, Dalian, China). Briefly, a mixture containing 1 µg of total RNA, 1 µL of 5’-CDS Primer A, and nuclease-free H_2_O to a final volume of 11 µL was denatured at 72°C for 10 min and then cooled to 42°C for 2 min. Subsequently, 1 µL of SMARTer II A Oligonucleotide was added. The first-strand cDNA synthesis was then performed in a total volume of 20 µL, containing the pre-denatured RNA-primer mixture, 4 µL of 5× First-Strand Buffer, 0.5 µL of DTT (100 mM), 1 µL of dNTP Mix (20 mM), 0.5 µL of RNase Inhibitor (40 U/µL), and 2 µL of SMARTScribe Reverse Transcriptase (100 U). The reaction was incubated at 42°C for 100 min, followed by enzyme inactivation at 72°C for 10 min. The resulting 5’-RACE-Ready cDNA was diluted with 240 µL of Tricine-EDTA Buffer and stored at -20°C for subsequent use.

PCR Amplification of the CDR3H Repertoire

The CDR3H repertoire was amplified by PCR using SeqAmp DNA Polymerase. Each 50 µL reaction contained 25 µL of 2× SeqAmp PCR Buffer, 2.5 µL of 5’-RACE-Ready cDNA, 2 µL of forward primer (TCACCARGGACAACTCCAAGA), 2 µL of reverse primer (ACACCAGGGGGAAGACTCTCGGG), and nuclease-free H_2_O to volume. The PCR was conducted under the following conditions: initial denaturation at 94°C for 30 s; 25 cycles of denaturation at 94°C for 30 s, annealing at 52°C for 30 s, and extension at 72°C for 1 min; with a final extension at 72°C for 5 min.

### 2.3 Sequencing and bioinformatics analysis

Library construction was performed using Illumina-compatible primers. Each 30 µL PCR reaction contained 15 µL of 2× Hieff^®^ Robust PCR Master Mix, 1 µL of barcoded forward primer, 1 µL of reverse primer, 10~20 ng of PCR amplicons, and nuclease-free H_2_O to volume. The reaction was gently mixed, briefly centrifuged, and amplified under the following program: 95°C for 3 min; 8 cycles of 94°C for 20 s, 55°C for 20 s, and 72°C for 30 s; followed by a final extension at 72°C for 5 min.

Library quality was assessed by 2% agarose gel electrophoresis for fragment size distribution and quantified using the Qubit^®^ 4.0 Fluorometer. PE300 paired-end sequencing (2 × 300 bp) was performed by Sangon Biotech (Shanghai, China).

IMGT/HighV-QUEST is the web portal of IMGT^®^ (24–27), the international ImMunoGeneTics information system^®^ (www.imgt.org for the analysis of rearranged nucleotide sequences of the antigen receptors (immunoglobulins (IG) or antibodies and T cell receptors (TR)) obtained from next generation sequencing (NGS) (28), based on IMGT-ONTOLOGY and the immunoinformatics IMGT scientific rules (29, 30).

Raw sequencing reads were processed using IMGT/HighV-QUEST, and high-quality productive sequences were retained for subsequent statistical analyses. The following analyses were performed:

(1) The germline V, D, and J gene segments used in the recombination of each read were identified, and their usage frequency and recombination diversity were statistically analyzed.
(2) Junctional diversity was analyzed based on the junction regions defined by IMGT/HighV-QUEST, and the CDR3H length was determined.
(3) Sequences were grouped according to CDR3H length distribution, and the length characteristics of P3’ V, P5’ J, P5’ D, P3’ D, N1, and N2 within each group were statistically characterized.
(4) Reads involved in the statistical analysis were classified into IgM1 and IgM2 groups based on nucleotide differences between the μ1 and μ2 genes. The length characteristics of P3’ V, P5’ J, P5’ D, P3’ D, N1, and N2 were statistically compared across and within the IgM1 and IgM2 groups, stratified by CDR3H length.
(5) The usage of D gene segments was assessed based on the lengths of the D gene fragments involved in recombination, which were obtained from IMGT/HighV-QUEST, compared with the lengths of the germline D gene segments published in IMGT.
(6) Graphs were generated and statistical analyses were performed using GraphPad Prism 6.

## 3 Results

### 3.1 Characteristics of the CDR3 region length distribution in the bovine IgH

**Figure 1:**
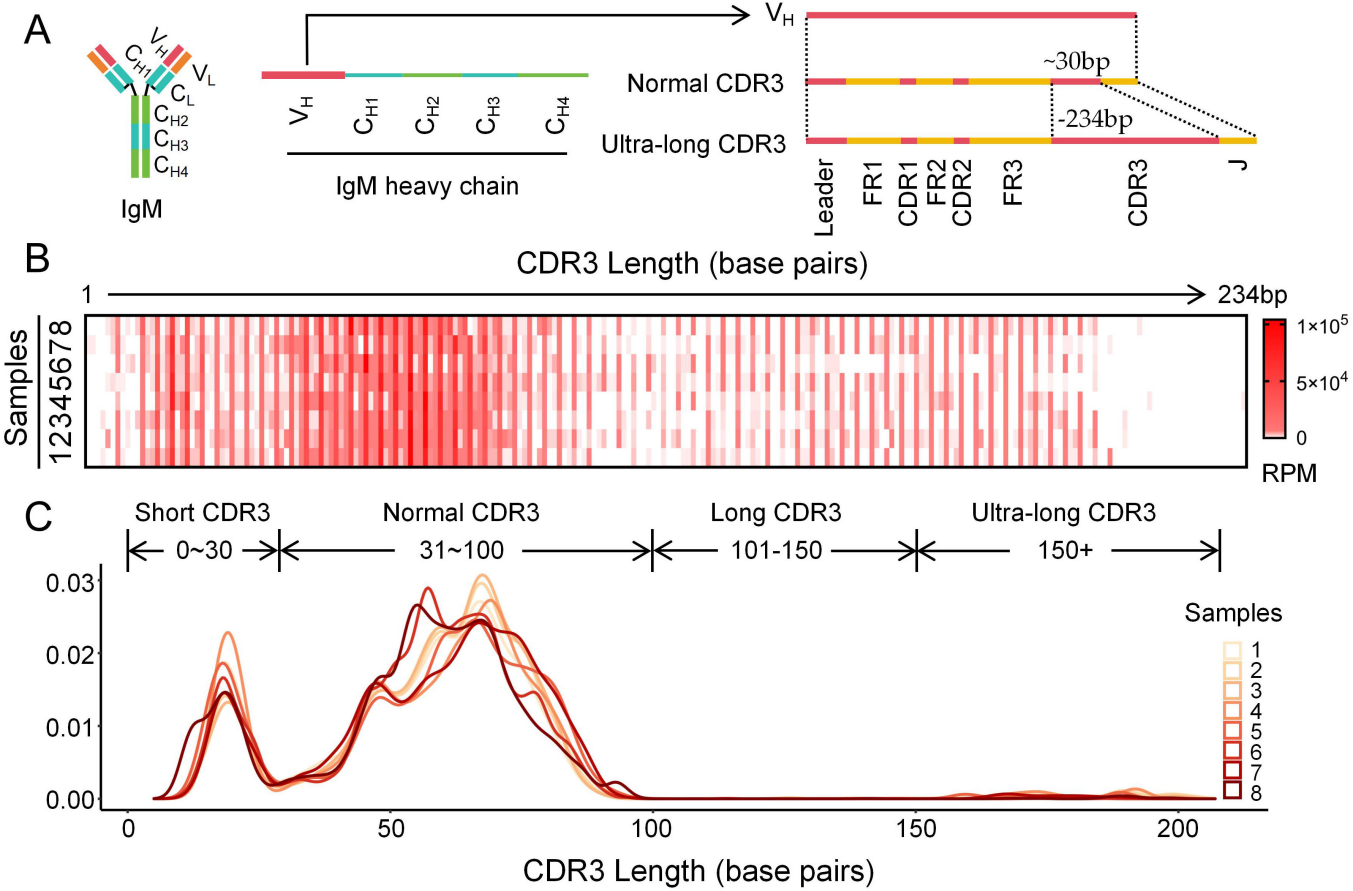
Features of the CDR3 region distribution in bovine IgH gene. **(A)** Diagram of the CDR3 region length distribution in bovine IgH gene; **(B)** The CDR3 length distribution in bovine IgH gene demonstrates a 3-base pair periodicity; **(C)** Reads density distribution of CDR3 regions with varying lengths in bovine IgH gene.

The CDR3 of the immunoglobulin heavy chain (IgH) was amplified and subjected to high-throughput sequencing using splenic tissues from eight adult cattle. Based on structural integrity criteria for immunoglobulins, a total of 473,067 high-quality reads were obtained from the eight samples (corresponding to 66,984; 59,895; 43,428; 56,560; 78,605; 48,659; 65,210; and 53,726 reads per sample, respectively). The CDR3 length exhibited a broad distribution, ranging from 1 to 234 bp (**Figure 1A**). In accordance with the principle of biological economy, the length distribution displayed a marked 3 bp periodicity (**Figure 1B**). Furthermore, the CDR3 regions were classified into four distinct categories based on length: short (11.91%~16.93%), normal (81.51%~85.02%), long (0.12%~0.30%), and ultra-long (0.16%~2.76%) (**Figure 1C**).

### 3.2 VDJ recombination in bovine IgH CDR3 regions of different lengths

**Figure 2:**
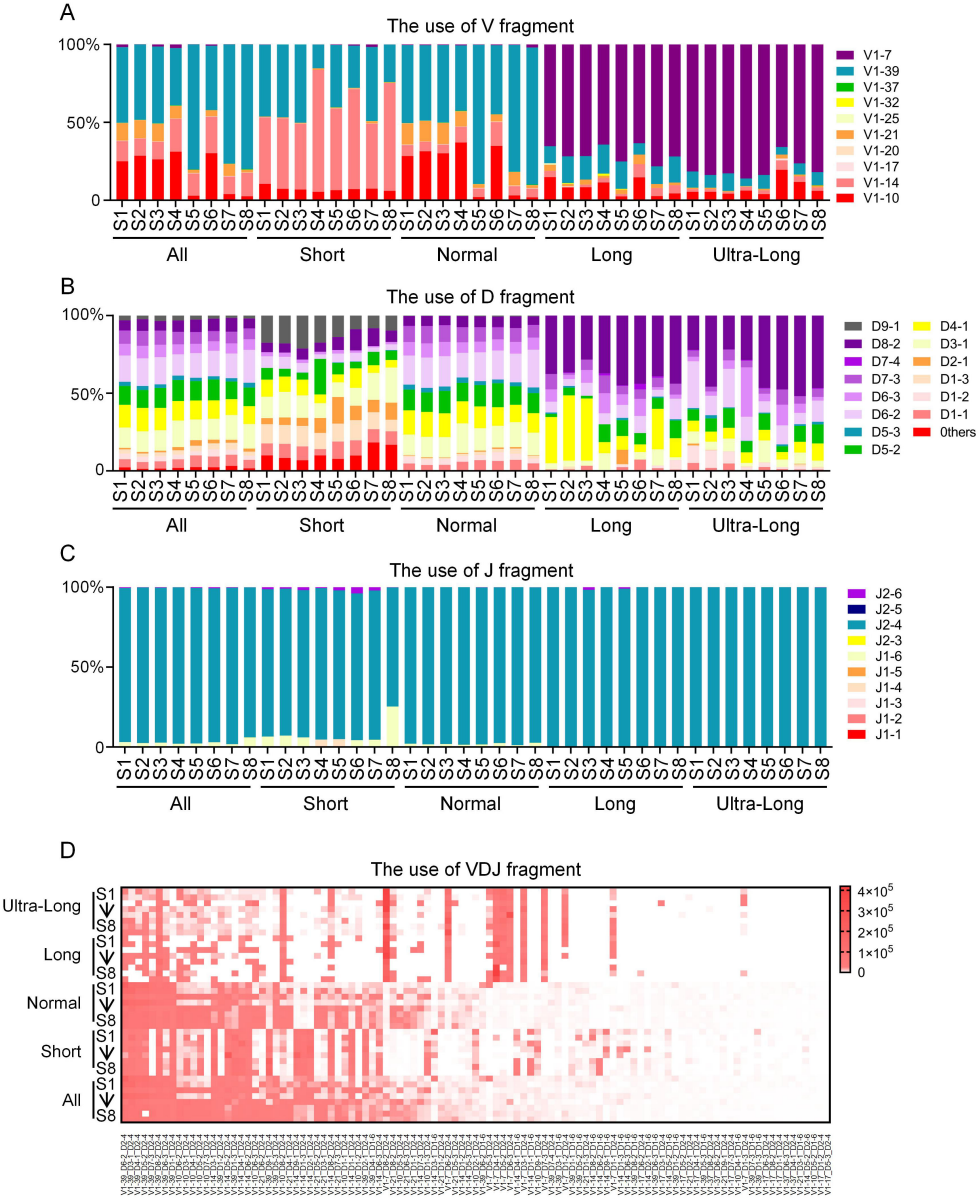
VDJ recombination in the different length CDR3 region of the bovine IgH gene. **(A)** V segments usage in the CDR3 region of the bovine IgH gene; **(B)** D segments usage in the CDR3 region of the bovine IgH gene; **(C)** J segments usage in the CDR3 region of the bovine IgH gene; **(D)** VDJ recombination characteristics in the CDR3 region of the bovine IgH gene. S1, sample1; S2, sample2; S3, sample3; S4, sample4; S5, sample5; S6, sample6; S7, sample7; S8, sample8.

To investigate the distinct patterns of VDJ recombination and gene segment usage across CDR3 length categories, sequences from eight cattle were analyzed based on the previously defined groups (short, normal, long, and ultra-long). Analysis revealed clear differences in V gene segment preference among the groups. The short CDR3 group was dominated by V1–14 and V1-39, the normal group was characterized predominantly by V1-39, while the long and ultra-long groups showed a strong preference for V1-7 (**Figure 2A**). These results indicate that the selection of specific V gene segments is closely associated with CDR3 length. D segment usage also varied considerably. While the short and normal groups exhibited broad diversity in D segment usage, the long and ultra-long groups demonstrated a significantly elevated frequency of D8-2 (**Figure 2B**). In contrast to the diversity of V and D segments, J segment usage was highly consistent across all groups, with J2–4 being the overwhelmingly dominant type (**Figure 2C**). The most frequent VDJ recombination types further highlighted the unique characteristics of each group. The most frequent recombination types identified in the short group were V1-39_D9-1_J2-4, V1-14_D3-1_J2-4, V1-39_D1-3_J2-4, etc. The most frequent recombination types identified in the normal group were V1-39_D6-2_J2-4, V1-39_D3-1_J2-4, V1-39_D4-1_J2-4, etc. The most frequent recombination types identified in the long group were V1-7_D4-1_J2-4, V1-7_D8-2_J2-4, V1-10_D8-2_J2-4, etc. The most frequent recombination types identified in the Ultra-long group were V1-7_D6-2_J2-4, V1-7_D8-2_J2-4, V1-7_D1-2_J2-4, etc (**Figure 2D**). These distinct recombination patterns underscore the specialization of the immunoglobulin repertoire in CDR3 regions of different lengths.

### 3.3 Junctional diversity in bovine IgH CDR3 regions of different lengths

**Figure 3:**
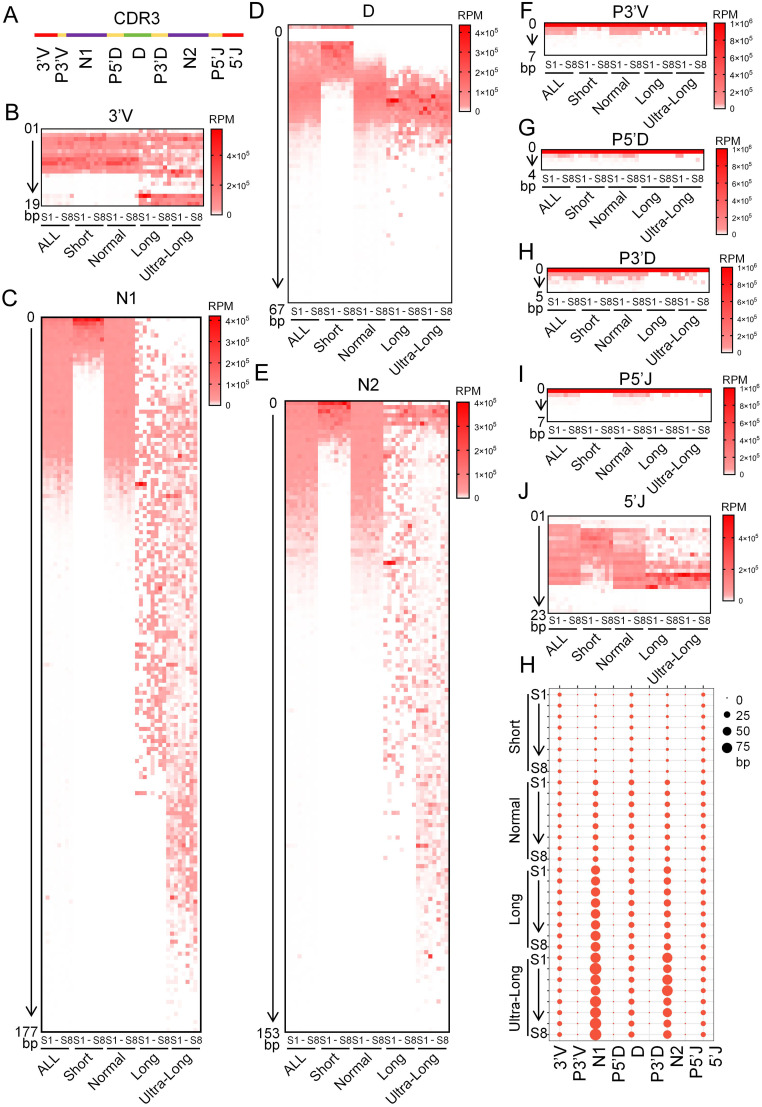
Junctional diversity in the different length CDR3 region of the bovine IgH. **(A)** Diagram of the Junctional diversity in bovine IgH CDR3 region; **(B–J)** The length distribution of the 3’V, P3’V, N1, P5’D, D, P3’D, N2, P5’J, and 5’J segments across CDR3 regions of different lengths in the bovine IgH gene; **(H)** The average length of the 3’V, P3’V, N1, P5’D, D, P3’D, N2, P5’J, and 5’J segments across CDR3 regions of different lengths in the bovine IgH gene. S1, sample1; S2, sample2; S3, sample3; S4, sample4; S5, sample5; S6, sample6; S7, sample7; S8, sample8.

The CDR3 region of the IgH gene is composed of nine segments, including 3’V, P3’V, N1, P5’D, D, P3’D, N2, P5’J, and 5’J (**Figure 3A**). The 3’V segment length within the bovine immunoglobulin heavy chain CDR3 region was stratified into two distinct patterns across the four CDR3 length groups: shorter 3’V lengths were observed in the short and normal-length groups, whereas longer 3’V lengths were associated with the long and ultra-long groups, suggesting that 3’V length influences the overall CDR3 length (**Figure 3B**). N nucleotide addition is a hallmark of the bovine IgH gene CDR3 region. The length of these N insertions increases with overall CDR3 length, establishing them as a key determinant of immunoglobulin diversity. Notably, the N1 region contributes more significantly to CDR3 length extension than the N2 region (**Figures 3C, E**). The D segment is an integral component of the IgH gene CDR3 region. In the short, normal, and long CDR3 groups, the actual utilized length of the D segment was positively correlated with the overall CDR3 length. However, despite a significant increase in CDR3 length from the long to the ultra-long group, the utilized D segment length did not show a corresponding increase (**Figure 3D**). P nucleotides contribute to the formation of the bovine immunoglobulin heavy chain CDR3 region. The addition of P3’V and P5’J nucleotides was more pronounced in the normal-length group compared to the other three groups. In contrast, the insertion lengths of P5’D and P3’ D showed no significant differences across all four groups (**Figures 3F–I**). The 5’J segment distribution exhibited considerable variation across bovine IgH CDR3 regions of different lengths. Its length progressively increased from the short to the long groups but did not increase further in the ultra-long group. This pattern indicates that the 5’J segment is a contributing factor to the overall CDR3 length in bovine IgH genes (**Figure 3J**). Collectively, diversity analysis of the CDR3 constituent segments revealed distinct contributions to length determination. N addition emerged as the principal determinant, with both N1 and N2 lengths increasing progressively with overall CDR3 length. N nucleotide addition emerged as the principal determinant, with both N1 and N2 lengths increasing progressively alongside the overall CDR3 length. Strikingly, while the utilized D gene length increased markedly from the short to the normal-length group, it plateaued thereafter and showed negligible change across the normal, long, and ultra-long groups. This indicates that the D segment is not the primary driver of extreme CDR3 elongation. Furthermore, although P nucleotide insertions exhibited some variation among the different CDR3 length groups, their contribution to the total length was minimal (**Figure 3H**).

### 3.4 Relationship between VD gene usage and junctional diversity in bovine IgH

**Figure 4:**
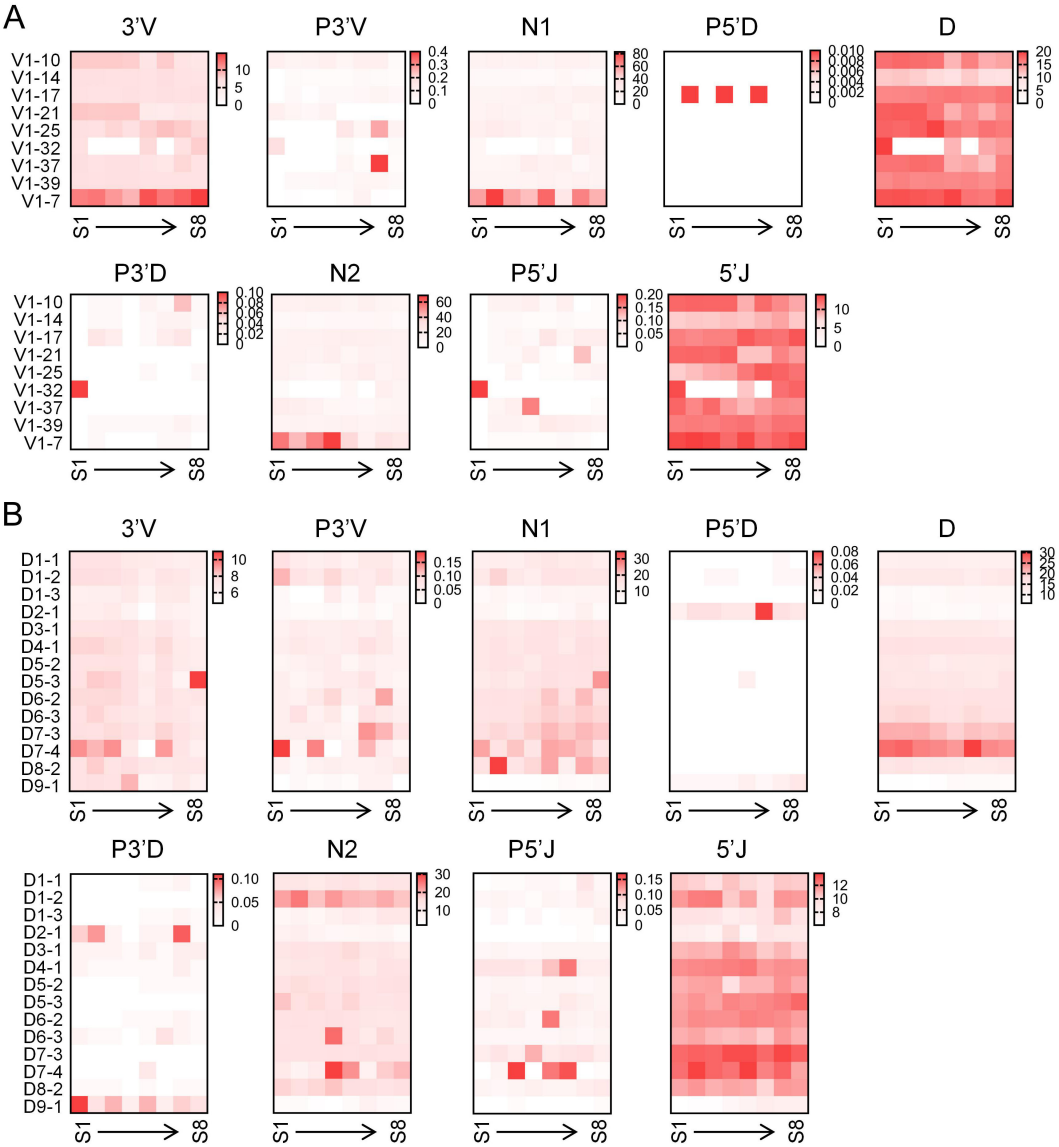
Relationship between V/D gene usage and junctional diversity in bovine IgH. **(A)** Relationship between V segments usage and junctional diversity in bovine IgH gene; **(B)** Relationship between D segments usage and junctional diversity in bovine IgH gene. S1, sample1; S2, sample2; S3, sample3; S4, sample4; S5, sample5; S6, sample6; S7, sample7; S8, sample8.

To investigate the relationship between V/D segment selection and junctional diversity in bovine IgH genes, we quantified the length of each CDR3 component associated with different V/D segments. The utilization of V1–7 was associated with a significantly longer 3’V segment, which can likely be attributed to the inherently longer 3’ end of the V1–7 gene compared to other VH segments. Furthermore, CDR3 regions employing V1–7 exhibited longer N1 and N2 nucleotide additions. In contrast, the lengths of P nucleotide additions and D gene segments showed no significant differences across the various VH genes used (**Figure 4A**). The utilization of D7–3 and D7–4 segments was associated with a longer utilized D segment length and more extensive P nucleotide additions at the 3’ D end compared to other D segments. Notably, despite these features, D7–3 and D7–4 do not generate the longest CDR3 regions in the bovine IgH locus, and their utilized length constitutes only a small fraction of their full genomic sequence. In contrast, when D9–1 was used, CDR3 regions exhibited markedly less N nucleotide addition and a shorter utilized D segment length. This characteristic may contribute to the formation of short CDR3 regions (**Figure 4B**).

### 3.5 VDJ recombination in the different length CDR3 region of the bovine IgM1 and IgM2

**Figure 5:**
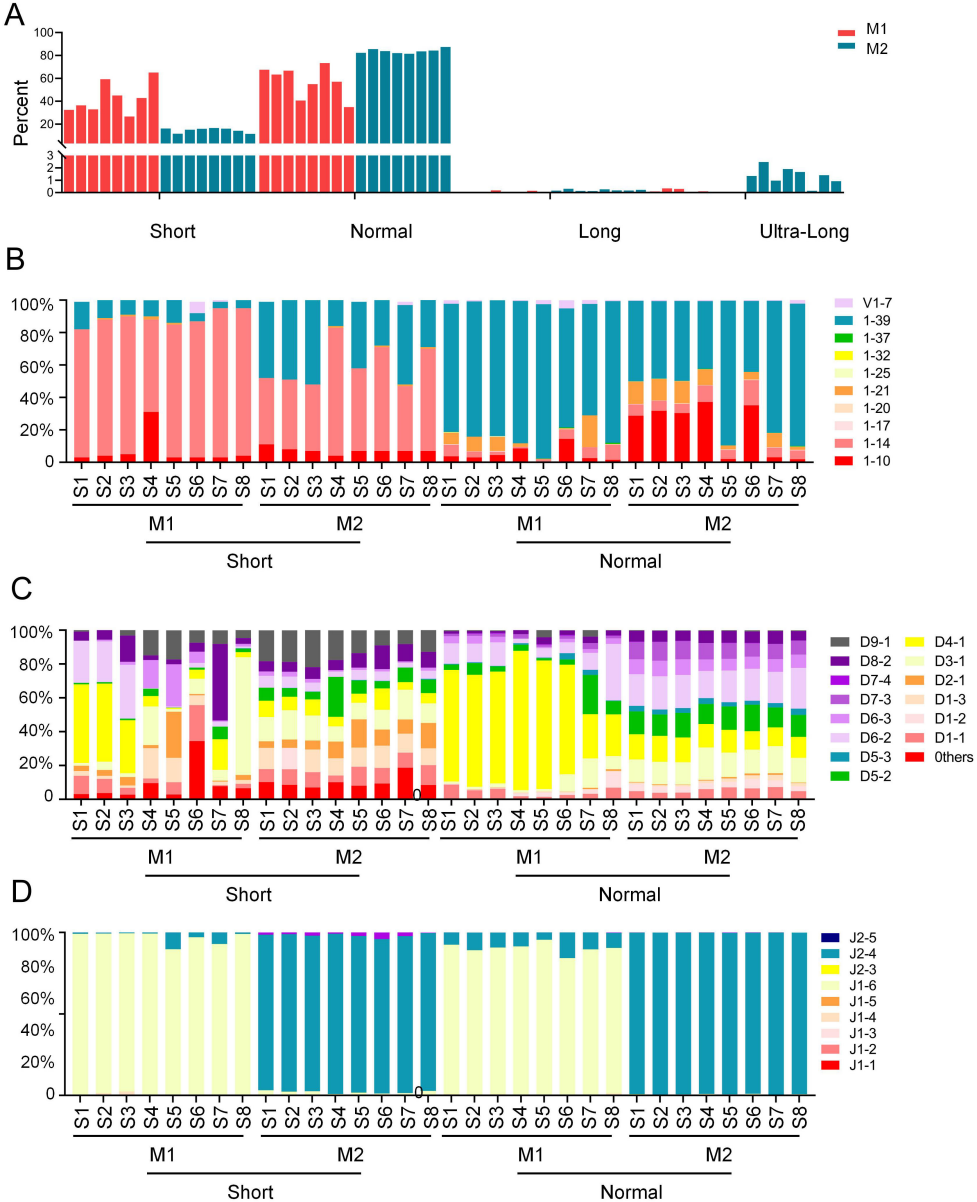
VDJ recombination in the different length CDR3 region of the bovine IgM1 and IgM2. **(A)** Reads density distribution of CDR3 regions across short and normal-length CDR3 regions in bovine IgM1 and IgM2 gene; **(B)** V segment usage in bovine IgM1 and IgM2 genes across short and normal-length CDR3 regions; **(C)** D segment usage in bovine IgM1 and IgM2 genes across short and normal-length CDR3 regions; **(D)** J segment usage in bovine IgM1 and IgM2 genes across short and normal-length CDR3 regions. S1, sample1; S2, sample2; S3, sample3; S4, sample4; S5, sample5; S6, sample6; S7, sample7; S8, sample8.

Bovine possess two IgM genes, designated as IgM1 and IgM2. To assess the relationship between CDR3 length and the usage of these isotypes, we stratified the amplified IgH gene CDR3 regions by both IgM isotype and CDR3 length. Analysis of the read length distribution revealed that IgM1 predominantly generates short and normal-length CDR3 regions, whereas long and ultra-long CDR3 regions were almost exclusively associated with IgM2 (**Figure 5A**). In the short CDR3 group, IgM1 usage was enriched for V1–14 and depleted for V1-39. In the normal group, the overall usage frequency of V1–39 was significantly increased compared to the short group, with IgM2 notably utilizing more V1-10 (**Figure 5B**). Both IgM1 and IgM2 genes exhibited a diverse D segment repertoire in the short CDR3 group. In the normal-length group, the D4–1 segment predominated in IgM1, whereas the diversity of D segments was significantly higher in IgM2 (**Figure 5C**). The IgM1 and IgM2 genes exhibit distinct preferences for J segment usage. IgM1 predominantly utilizes J1-6, whereas IgM2 primarily employs J2-4. This characteristic pattern is consistent across both the short and normal-length CDR3 groups (**Figure 5D**).

### 3.6 Junctional diversity in short and normal-length CDR3 regions of bovine IgM1 and IgM2

**Figure 6:**
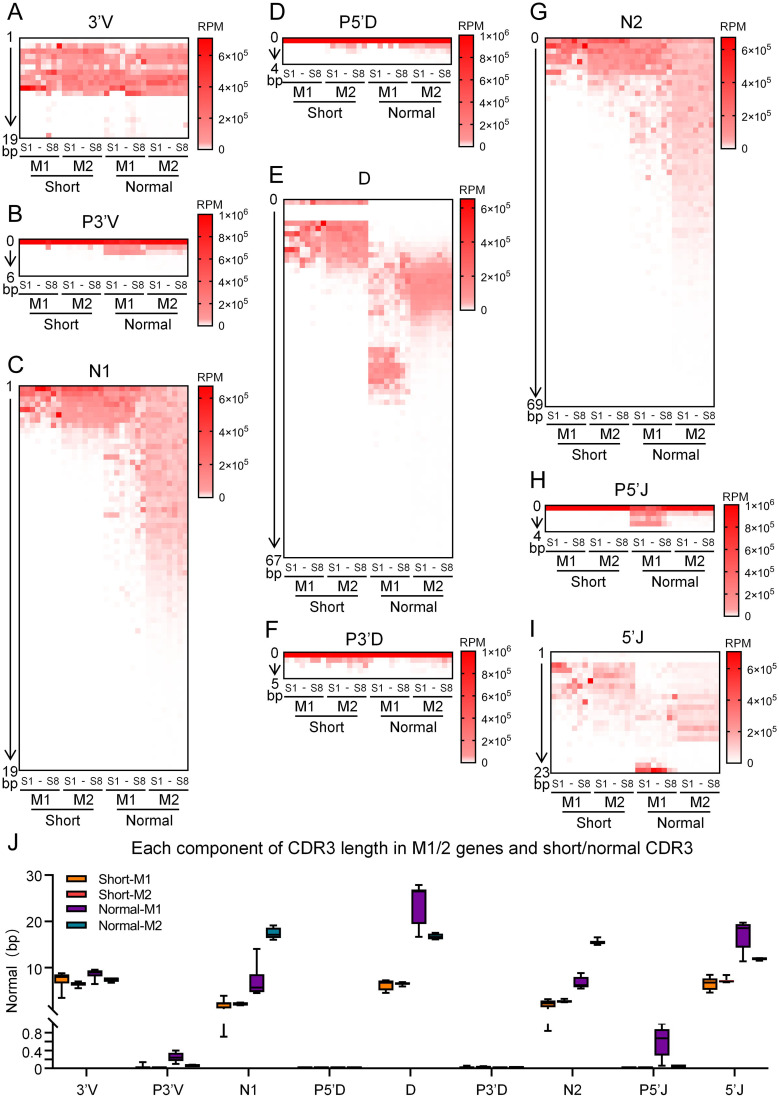
Junctional diversity in short and normal-length CDR3 regions of bovine IgM1 and IgM2. **(A–I)** The length distribution of the 3’V, P3’V, N1, P5’D, D, P3’D, N2, P5’J, and 5’J segments was analyzed across short and normal-length CDR3 regions in bovine IgM1 and IgM2 genes.; **(J)** A comparative analysis of the average segment lengths was conducted between short and normal-length CDR3 regions in the bovine IgM1 and IgM2 genes. S1, sample1; S2, sample2; S3, sample3; S4, sample4; S5, sample5; S6, sample6; S7, sample7; S8, sample8.

To assess the relationship between different IgM genes and CDR3 junctional diversity, we classified the reads of bovine IgH genes into four groups: IgM1-short, IgM2-short, IgM1-normal, and IgM2-normal. The length distribution of each component in the CDR3 regions was analyzed across these groups. The results demonstrated that the 3′V length remained largely consistent among all four groups (**Figure 6A**). The addition of P3′V nucleotides was similar between IgM1 and IgM2 in both the short and normal-length groups (**Figure 6B**). In the short group, the lengths of N1 and N2 nucleotides were comparable between IgM1 and IgM2. However, in the normal-length group, IgM1 exhibited shorter N1 and N2 nucleotides compared to IgM2 (**Figures 6C, G**). The actual length of D segments was similar between IgM1 and IgM2 in the short group but longer in IgM1 than in IgM2 within the normal-length group (**Figure 6E**). No significant differences were observed in the addition of P5′D or P3′D nucleotides among the four groups (**Figures 6D, F**). In the normal-length group, IgM1 showed more extensive P5′J nucleotide additions than IgM2 (**Figure 6H**). Additionally, the length of the 5′J segment was significantly greater in IgM1 compared to IgM2 in the normal-length group (**Figure 6I**). In the normal group, the IgM1 gene possessed longer 3’V, P3’V, D, P5’J, and 5’J segments, but shorter N1 and N2 segments compared to IgM2 (**Figure 6J**).

### 3.7 Relationship between D segments usage and junctional diversity in bovine IgM1 and IgM2

**Figure 7:**
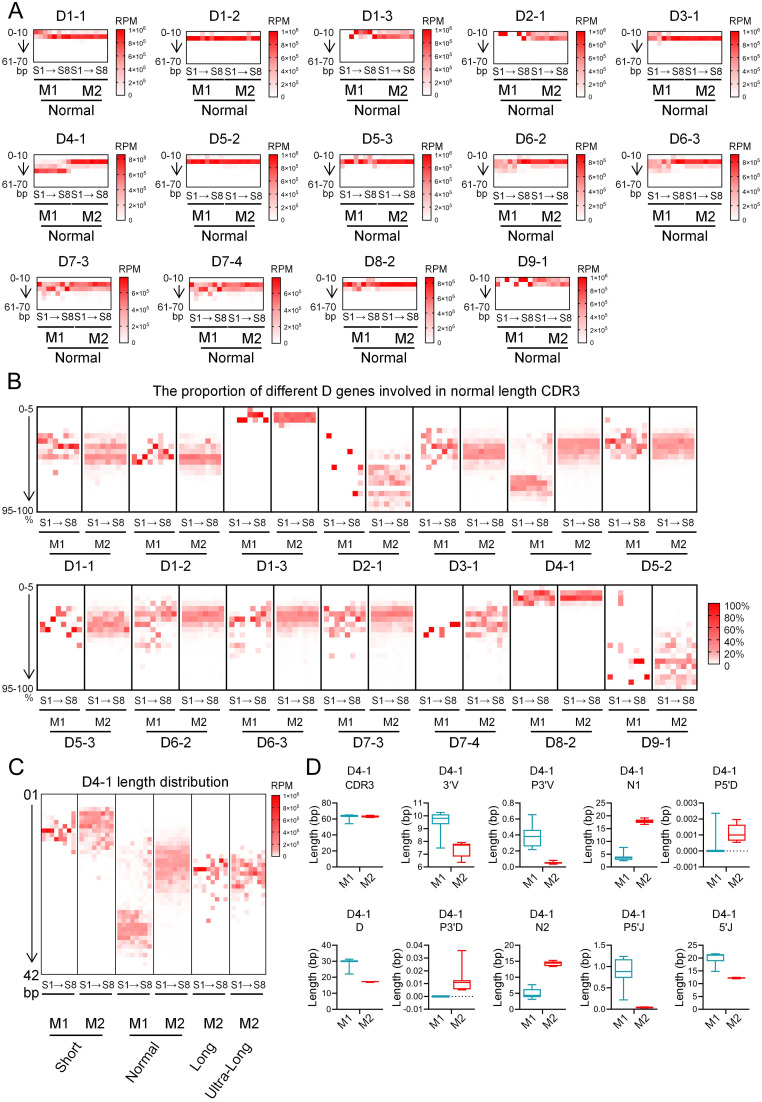
Relationship between D segments usage and junctional diversity in bovine IgM1 and IgM2. **(A)** Actual utilized length of D segments within the normal-length CDR3 region in bovine IgM1 and IgM2; **(B)** The percentage of the actual utilized length of D segments relative to their genomic length in the normal-length CDR3 region of bovine IgM1 and IgM2. **(C)** Actual utilized length of D4–1 segments within the different length CDR3 region in bovine IgM1 and IgM2; **(D)** Junctional diversity of CDR3 regions containing D4–1 in bovine IgM1 and IgM2. S1, sample1; S2, sample2; S3, sample3; S4, sample4; S5, sample5; S6, sample6; S7, sample7; S8, sample8.

To investigate whether the characteristic utilized length of D segments in the normal-length CDR3 regions of different IgM genes is associated with the usage of specific D gene segments, we analyzed the normal-length group according to IgM gene type and D segment usage, and further evaluated the utilized length of the D segments. The results showed that although the genomic lengths of D gene segments vary considerably, their utilized lengths remained within a range of approximately 10~30 bp across different D segments. Interestingly, when D4–1 was used, the utilized D segment length in IgM1 was significantly longer than that in IgM2 (**Figure 7A**). Due to the substantial variation in D segment genomic length, we further analyzed the utilization percentage of each D segment. The results revealed that the usage percentage of D4–1 was markedly higher in IgM1 than in IgM2, making it the highest among all D segments except D9-1 (**Figure 7B**). Further analysis of the distribution of D4–1 across different CDR3 length groups and IgM genes showed that in the short group, the utilized length of D4–1 was short in both IgM1 and IgM2, with no significant difference between the two. In the normal-length group, however, the utilized length of D4–1 in IgM1 was significantly longer than that in IgM2. In the long and ultra-long groups, the utilized length of D4–1 was consistent between the two IgM types but notably shorter than that of IgM1 in the normal-length group (**Figure 7C**). In the normal-length group, the overall CDR3 length was similar between IgM1 and IgM2. Despite this consistency in total length, the composition of CDR3 components differed considerably: compared to IgM2, IgM1 exhibited longer 3′V, P3′V, D, P5′J, and 5′J segments, but shorter N1, P5′D, P3′D, and N2 segments (**Figure 7D**).

## 4 Discussion

The immunoglobulin CDR3 is a region of core value within the antibody molecule, with its importance stemming from its irreplaceable role in antigen recognition and its unique status as a key marker of immune system diversity and adaptability. Located at the center of the antibody variable region, CDR3 is formed through a complex rearrangement process of the V(D)J gene segments of the heavy and light chains. The unique mechanisms of this process-junctional diversity (random insertion and deletion of nucleotides) and somatic hypermutation-endow CDR3 with unparalleled sequence diversity and structural variability, making it the most critical “structural fingerprint” that determines the specificity and affinity of antigen-antibody binding. CDR3 not only serves as the central interface where the BCR directly contacts and anchors antigenic epitopes, but its overall sequence repertoire also constitutes an individual’s BCR immune repertoire (31). This enables a dynamic and comprehensive reflection of the immune system’s health status, diversity levels, and response history to infections, vaccinations, or autoantigens.

A critical consideration in repertoire studies is the choice of immunoglobulin isotype. While ultra-long CDR3 regions have been reported in switched isotypes like IgG (14, 18), we specifically targeted the IgM repertoire to achieve the primary objective of this study: to define the baseline characteristics and generative mechanisms of the naïve B cell repertoire. IgM is the first isotype expressed during B cell development and dominates the pre-immune repertoire. By analyzing IgM, we capture the diversity landscape immediately after V(D)J recombination, thus providing a clear view of the initial molecular events—including V(D)J segment choice and junctional processing—that give rise to the full spectrum of CDR3 lengths, before these processes are confounded by antigen-driven selection, class switching, and extensive somatic hypermutation.

While the bovine antibody repertoire has garnered significant interest due to the presence of antibodies with exceptionally long CDR3 regions, previous investigations have predominantly focused on elucidating the formation mechanism of these ultra-long CDR3s in isolation. In contrast, our study provides a comprehensive and systematic characterization of the entire bovine IgH CDR3 repertoire, establishing a complete spectrum of CDR3 lengths—classified into short, normal, long, and ultra-long categories. This approach allows us to move beyond a singular focus on an extreme phenotype and instead uncover the fundamental organizational principles and distinct molecular architectures that differentiate these length-based categories. We demonstrate that each category is associated with a unique VDJ recombination signature and that the contribution of junctional diversification mechanisms, particularly N-addition, varies substantially across the repertoire. Furthermore, our discovery of a functional division of labor between the IgM1 and IgM2 isotypes, with IgM2 being almost exclusively responsible for generating long and ultra-long CDR3s. Thus, this work shifts the paradigm from studying the ultra-long CDR3 as a curiosity to understanding the bovine antibody repertoire as a holistically structured and highly compartmentalized system.

Multiple high-throughput sequencing studies have revealed that bovine IgH CDR3 fragments exhibit an exceptionally broad length span, ranging from approximately 1 bp to over 204 bp (12, 21). This unique length distribution pattern contrasts sharply with the relatively concentrated and continuous CDR3 length distributions observed in humans and mouse. In this study, the longest CDR3H identified in the recombinant sequences reached 234 base pairs, surpassing all previously reported ultra-long CDR3H sequences, through expanded sample collection and greater sequencing depth. Density stacking analysis of all CDR3H lengths revealed a distinct 3-bp periodicity and four distinct segments. This 3-bp periodicity in CDR3H has also been observed in swine immunoglobulins (32, 33). Existing studies have not provided a clear definition for the classification of CDR3 lengths, and the criteria for defining ultra-long CDR3 vary across the literature (12, 18, 34). Therefore, based on approximately 500,000 reads subjected to CDR3 length density stacking, we identified a four-segment distribution of CDR3 lengths: 0~30 bp, 31~100 bp, 101~150 bp, and >150 bp (**Figure 1C**). Among these, the short group accounted for 11.91%~16.93%, the normal group accounted for 81.51%~85.02%, while notably, the long group constituted only 0.12%~0.30%, significantly lower than the ultra-long group at 0.16%~2.76%. To prevent the high frequency of the normal group from obscuring the characteristics of the other three CDR3 groups, subsequent analyses were conducted separately for the four groups to elucidate the compositional features of CDR3H across different length categories. It is worth noting that even the so-called “normal-length” CDR3 in cattle (31–100 bp) substantially exceeds the CDR3 lengths of species such as mice, humans, and sheep (around 50 bp). Moreover, cattle stably exhibit recombinant forms with CDR3 lengths exceeding 100 bp. Thus, cattle represent a highly valuable unique model for studying the contribution to immunoglobulin CDR3 length diversity.

Recombination diversity analysis of the four groups revealed distinct usage preferences among CDR3H of different lengths. The long and ultra-long groups preferentially used VH1-7, consistent with previous studies on bovine ultra-long CDR3H (18, 21). However, different VH usage preferences were observed in the short and normal groups: the short group favored V1–14 and V1–39 for recombination, while the normal group predominantly used V1-39. D8–2 was preferentially used in the long and ultra-long groups, whereas D4–1 was more frequently observed in the short and normal groups compared to the long and ultra-long groups.

Further composition analysis of bovine CDR3H revealed significant differences in the contributions of different regions to CDR3H across the groups. The 3’V segments in the long and ultra-long groups were significantly longer than those in the short and normal groups. This may be associated with the preferential usage of V1–7 in the long and ultra-long groups. The 3’V of V1–7 is markedly distinct from that of other V genes, and its 3’ end sequence “actactgtgcaccaga” is considerably longer than that of other V genes (12, 18, 21). The length of the P3’V segment was particularly prominent in the normal group. The selection of cleavage sites by the Artemis-DNA-PKcs complex determines the length of P3’V, which is directly influenced by the cleavage position of the Artemis nuclease on the hairpin structure at the end of the V gene. The sequence of the hairpin stem governs its thermal stability and structure; hairpin stems rich in A-T base pairs are more flexible, potentially leading to more dispersed Artemis cleavage sites and greater variability in P3’V length (35–37). Similarly, the 5’J segment length was significantly longer in the normal group compared to the other three groups. While the contribution of the D segment to junctional diversity is established, a positive correlation between D segment length and CDR3H length was only observed in the short and normal groups; no significant variation in D segment length was noted in the long and ultra-long groups. The length distribution of N nucleotides exhibited the greatest diversity among all factors, with N1 contributing more strongly to CDR3 than N2. Unlike the significant role of N nucleotides in other groups, the primary contributors to CDR3 in the short group were 3’V, D, and 5’J. In the normal group, N1, D, and N2 contributed comparably to CDR3. In the long and ultra-long groups, N1 contributed the most to CDR3, followed by N2, which may be related to the V-DJ and D-J recombination processes (**Figure 3H**). The finding that N-nucleotide addition is a major contributor to the length of long and ultra-long CDR3s appears to contrast with several key studies that emphasize germline D segment-mediated mechanisms. Similarly, an internal duplication in the IGHV1–7 gene and minimal N-diversity at the V-D/D-J joint in ultra-long CDR3s were identified. Similarly, conserved short nucleotide sequences and templated duplications have been identified, suggesting mechanisms other than random N-addition dominate junctional diversity (19, 21, 23). We propose that these seemingly divergent conclusions can be harmonized by considering the distinct analytical focuses and the multi-faceted nature of CDR3 generation. The discrepancy likely arises from algorithmic focus in segment assignment. Bioinformatic tools can differ in how they prioritize the alignment of sequences to germline genes. Algorithms like BLASTN excel at identifying high-similarity regions and may optimally align sequences by assigning more nucleotides to the germline V, D, or J segments. In contrast, the IMGT/HighV-QUEST platform used in our study employs a standardized ontology that rigorously defines the boundaries of V, D, and J genes, often resulting in a more conservative assignment of germline identity and a consequent attribution of more sequence to non-templated N-regions.

Grouped analysis based on different V and D genes revealed that when V1–7 was selected for recombination, the lengths of 3’V, N1, and N2 were longer compared to other groups. When D7–2 and D7–3 were used, the length of the D segment involved in recombination was longer than in other groups, despite the germline genomic lengths of D7–3 and D7–4 being only 67 bp and 73 bp, respectively, not the longest among D gene segments. This indicates less trimming of the germline segment during recombination when D7–2 and D7–3 are used. In contrast, D1–3 and D8-2, which have germline gene segment lengths exceeding 100 bp, underwent substantial trimming during recombination. This may be consistent with the presence of conserved motifs encoded at the 5’ and 3’ ends, such as the CPDG turn motif at the 5’ end and the alternating aromatic amino acids YxYxY at the 3’ end (21). To investigate whether CDR3 length distribution correlates with the μ gene, the dataset was further stratified by μ gene. The μ1 gene primarily recombined with short and normal length CDR3, whereas the long and ultra-long groups almost exclusively selected the μ2 gene. To analyze whether the selection of CDR3 length by different μ genes is associated with V gene usage, short-M1 predominantly used VH1–14 for recombination, at a much higher frequency than short-M2. Similarly, normal-M1 used VH1–39 at a significantly higher frequency than normal-M2. An analogous preference was observed for DH segments. D4–1 appeared in normal-M1 at a much higher frequency than in other groups. Composition analysis of CDR3 in these four groups yielded more intriguing findings: the M1 group exhibited significantly longer P3’V, D, P5’J, and 5’J segments than the M2 group, but notably shorter N1 and N2 segments. Thus, CDR3 length also correlates with the μ gene.

The selective trimming of D genes is a focus of particular interest. Although ultra-long D genes participate in recombination, their contribution to CDR3 length does not show substantial variation, primarily ranging between 10~30 bp. A remarkable 47.6% of ultralong CDR3 sequences contain in-frame nucleotide deletions within the IGHD8–2 region, as independently discovered through a different methodological approach, providing strong corroboration for this phenomenon (Stanfield et al., 2019). In the normal group, the usage frequency of D4–1 differed between M1 and M2, while the usage percentages of the ultra-long D1–3 and D8–2 were significantly lower than other D gene segments, and the short segment D9–1 had the highest usage percentage. The extensive deletions in D8–2 may resemble the resection mechanism of failed Class Switch Recombination (CSR), potentially resulting from AID-mediated double-strand breaks. Concurrently, the presence of numerous hotspot mutation motifs within the D8–2 segment suggests that these events may be attributed to strand slippage associated with AID activity (38–40). Therefore, stochastic deletions during recombination are associated with the D gene. Further analysis of the lengths of CDR3 component segments in D4–1 M1 and M2 groups revealed that the 3’V, 5’J, and D segments were relatively intact in the M1 group, whereas the P3’V, P5’D, P3’D, and P5’J segments were extremely short, and N1 and N2 were substantially lower than in other groups. At the junctions, there is competition between TdT activity and exonuclease activity. In most cases, this system maintains a balance, with moderate trimming and limited N-additions being the norm. If exonuclease activity dominates, segments become shorter; if TdT activity dominates, N-regions lengthen. Recombination involving D4–1 appears to represent a shift in this balance towards the “conservative” end. Multiple fragments longer than 50 bp were found in the D gene segments of the bovine immunoglobulin heavy chain gene locus structure. The expressed D genes of the bovine immunoglobulin heavy chain also have a certain degree of diversity, but the actual length of the D fragments is relatively stable, mostly ranging from 20 to 30 bp (21). In contrast, the length of the D fragments of the bovine IgH gene is rich and diverse, and its usage rate is lower than that of most species (21, 41–43). The identification of AID-driven deletion events as a major diversifying force within the ultralong CDR3 repertoire powerfully illuminates the mechanism underlying this discrepancy—why bovine IgH possesses long germline D fragments but extensively trims them during recombination (Stanfield et al., 2019). Unraveling the underlying mechanism will provide some new perspectives for enriching the research on artificial antibody preparation, especially in the preparation of antibodies related to easily mutating viruses.

Terminal deoxynucleotidyl transferase (TdT) exhibits a distinct substrate preference for dGTP and dCTP during catalysis, and competition from DNA repair/ligation mechanisms may restrict the duration of TdT activity, potentially explaining why N1 is longer than N2 (44, 45). Our findings, which highlight the predominant role of N-addition in shaping CDR3 length, invite consideration of how this process might be regulated throughout ontogeny. It is well-established in mammals that TdT activity is low in neonates, resulting in repertoires with fewer N-additions and shorter CDR3 regions (46, 47). It was demonstrated that the bovine IGHV1S1 (BF4E9) and IGHD2 (DH2) genes, which are known to encode antibodies with exceptionally long CDR3H, are already preferentially expressed in B cells at birth (46, 47). This presents a fascinating paradox: the molecular machinery favoring the generation of ultra-long CDR3s (i.e., specific V/D genes) is active early in life, while the primary enzymatic driver of length extension (TdT) is subdued. This suggests that the full potential for generating ultra-long CDR3s, as characterized in our adult repertoire, is likely attained postnatally as TdT expression increases. Therefore, we propose that the bovine antibody repertoire undergoes a dynamic maturation process, where the neonatal repertoire is shaped by strong germline-encoded biases, which is subsequently diversified and elongated by high TdT activity in adults. Future analysis of neonatal repertoires will be crucial to test this hypothesis and to fully understand the developmental regulation of this unique antibody architecture.

In conclusion, we propose that the formation of CDR3H length diversity in cattle is complex, arising not only from recombination preferences but also regulated by factors such as the balance between exonuclease and TdT activities, specialized domains within gene segments, N and P nucleotide additions, stochastic trimming of germline gene segments, and the formation of unique structures and disulfide bond patterns, all collectively contributing to enhanced CDR3 diversity.

## Data Availability

The datasets presented in this study can be found in online repositories. The names of the repository/repositories and accession number(s) can be found in the article/supplementary material.
